# Validation of the caregiver skills (CASK) scale in a Dutch sample of carers for adolescents with eating disorders

**DOI:** 10.1186/s40337-026-01561-6

**Published:** 2026-03-02

**Authors:** C. M. T. Schilder, U. N. Danner, D. J. Hessen, A. A. van Elburg, L. C. Sternheim

**Affiliations:** 1 Altrecht Eating Disorders Rintveld, Zeist, The Netherlands; 2 Department of Clinical Psychology, Utrecht University, Utrecht, The Netherlands; 3 Department of Methodology and Statistics, Utrecht University, Utrecht, The Netherlands

**Keywords:** CASK, Anorexia nervosa, Caregiver skills, Caregiver support programs, Eating disorders, Validity, psychometric properties, Questionnaire validation.

## Abstract

**Background:**

Carers are crucial to the recovery process of people with eating disorders (EDs). The Caregiver Skills (CASK) scale was developed to assess key caregiving skills among carers of individuals with EDs. While psychometric evaluations of the English, German and Spanish versions have supported the original six-factor model, no validated Dutch version exists to date. This study aimed to examine the psychometric properties of a Dutch translation of the CASK. Validated caregiving outcome measures can assist in exploring coping skills in clinical practice and support research evaluating both caregiving interventions and ED treatments.

**Methods:**

A total of 248 carers (139 female, 109 male; mean age = 48.3 years) completed the CASK prior to assessment at a specialized ED treatment center in the Netherlands. The internal consistency of the total scale and six subscales was assessed using McDonald’s omega. Confirmatory factor analysis (CFA) was used to test the original six-factor structure. Gender differences in subscale and total scores were explored using linear mixed-effects models (LMM).

**Results:**

The original six-factor structure could not be confirmed in CFA, and model fit remained suboptimal after post hoc modifications. However, CFA results for the six subscales separately showed good to acceptable fit, and internal consistency was satisfactory. No gender differences were found in total CASK scores, though male carers reported higher skills on ‘Self-Care’ and ‘Insight and Acceptance’, and female carers reported higher skills on ‘Frustration Tolerance’ and ‘Emotional Intelligence’.

**Conclusions:**

The Dutch version of the CASK did not replicate the original six-factor structure at the full-scale level. Nonetheless, the six subscales demonstrated adequate psychometric properties and appear particularly suitable for use at the group level, making the instrument relevant for research and care improvement policies. Further studies are needed to determine whether the CASK can also provide added value at the individual level, for example to guide individualized treatment content, and to evaluate its applicability in more diagnostically and culturally diverse samples. While male and female carers reported comparable overall skills, differences in specific subscale scores highlight the importance of considering gender in the context of skill development and support for carers of individuals with EDs.

**Supplementary Information:**

The online version contains supplementary material available at 10.1186/s40337-026-01561-6.

## Abstract (plain language summary)

The goal of this study was to test how well the Dutch version of the Caregiver Skills (CASK) questionnaire works for people who support someone with an eating disorder. The CASK measures key caregiving skills, such as setting boundaries, coping with emotions, communicating effectively, and taking care of one’s own wellbeing. We asked 248 caregivers from a specialist treatment center in the Netherlands to complete the CASK. We found that the overall structure seen in other countries did not fully fit the Dutch data, but each skill area worked well on its own. On average, male and female carers had similar overall skill levels, though male carers reported stronger Self-Care and Insight and Acceptance, while female carers scored higher on Frustration Tolerance and Emotional Intelligence. These findings can help improve caregiver support programs.

**Supplementary Information:**

The online version contains supplementary material available at 10.1186/s40337-026-01561-6.

## Introduction

Carers (i.e. parents, family, friends) play a vital role in the recovery process of individuals with an eating disorder (ED), particularly adolescents [1]. They often spend substantial time and effort providing both practical support such as financial help, arranging treatment, transport, navigating care systems and transitioning from adolescent to adult services [2–4]. Carers also provide emotional support, in managing meal times, ED behaviours and negative emotions of their child [2, 3]. When the person with the ED is very ill, carers find themselves having to take over authority and daily decision making.

Given their vital role in recovery, carers of individuals with ED require strong caregiving skills, including adaptive coping, illness understanding, and emotional intelligence. However, carers often feel inadequate in managing these complex tasks and express a need for more guidance [4, 5]. Importantly, caregiving skills are an important predictor for caregivers’ burden [4], which tend to be very high in ED carers [6]. Moreover, improvements in caregiving skills have been associated with better ED treatment outcomes [7]. It is thus crucial to develop and evaluate interventions for carers that help improve caregiving skills.

The importance of carers’ involvement in ED treatment is emphasized in international clinical guidelines [8]. In evidence-based treatments for adolescents, such as family-based treatment (FBT) [9] and multi-family therapy (MFT) [10] carers initially take responsibility for restoring healthy eating patterns, achieving weight restoration where needed, and reducing ED behaviours such as binging and purging [11]. As treatment progresses, more autonomy is given to the child, but carers remain actively involved in managing mealtimes and behaviours and continue to participate in treatment sessions [11]. Carers are thus trained as co-therapists, with a focus on family strengths [12]. It has been posited that the success of the treatment stems from its focus on changing the way a family responds to and manages their child’s ED. Similarly, guidelines for adult EDs encourage carer involvement, for example in the Maudsley Model of Anorexia Nervosa Treatment for Adults [8, 13]. In addition, a growing number of carer-focused interventions—including self-help materials, groups, and telephone coaching—aim to provide psychoeducation, skills training, behavioural strategies, and peer support [1, 14, 15]. These approaches not only reduce carer distress and burden but can also benefit patient outcomes [1].

Although FBT and MFT are well researched, outcomes usually focus on patients’ recovery, with limited attention to whether these treatments improve caregiving skills or whether such improvements relate to patient outcomes. One study found reduced parental emotional over-involvement after FBT, with changes in expressed emotion of female carers explaining additional variance in anorexia nervosa (AN) symptomatology [14]. This suggests that improvements in caregiving skills may be important working mechanisms in ED treatment.

To evaluate their role in patient outcomes and the effectiveness of caregiver-focused interventions, suitable outcome measures are needed. The Caregiver Skills Scale (CASK) was developed as a brief self-report tool assessing key caregiving skills relevant to ED management [16]. The CASK is grounded in cognitive-interpersonal models of AN [13]. These models explain how unhelpful parental reactions, excessive parental emotions and dysfunctional parental communication styles may maintain AN symptoms. The CASK was designed to reflect domains such as emotional regulation, communication, self-care, and the ability to remain calm and supportive in difficult situations [16].

The CASK has been used to assess the effectiveness of caregiving interventions across countries. For instance, the German version of the CASK showed significant improvement in caregiver skills among Austrian carers after an 8-week workshop/online program and also after a 2-day multi-family intervention [17, 18]. A Dutch study of a caregiving intervention similarly found significant improvements on the total CASK score among carers of adolescents with (atypical) AN [19]. These findings align with UK studies that used the CASK to assess effectiveness of caregiving interventions in both adults and young people [20–23].

The original English CASK demonstrated strong psychometric properties, with high internal consistency for both the total scale and subscales, and exploratory and confirmatory factor analyses supporting a six-factor structure [16]. Convergent validity was shown through correlations with caregiver distress, expressed emotion, and accommodating behaviors. The scale also proved sensitive to change following skills-based interventions, with effect sizes varying from small to very large depending on intervention intensity [16]. After the publication of the CASK in English it was translated into the Spanish and German language. The Spanish version showed good psychometric properties and replicated the six-factor structure [24]. The German version of the CASK has demonstrated promising psychometric properties [24]. The six-factor structure was considered acceptable, and the scale showed strong internal consistency for both the total score and subscales. In addition, the German CASK demonstrated convergent validity through associations with caregiver distress, anxiety, depression, and expressed emotion. It also proved sensitive to change [24].

When examining the impact of caregiving skills on ED outcomes, it is important to note that most studies include more female than male carers and often do not differentiate between them in analyses [15]. Female carers may be overrepresented because they typically spend more time with the child, while male carers may find it harder to accept the ED as an illness and therefore participate less in treatment [25]. Yet the male carer’s parental role is important for the child’s recovery, and so is the development of coordinated care strategies between the carers [25]. Notably, levels of caregiver stress may vary significantly between male and female carers, and these levels may further vary depending on the specific phase of the child’s treatment [26]. Evidence on gender differences in CASK scores is mixed: Zeiler et al. [18, 24] found higher scores for male carers, whereas Vintró-Alcaraz [27] and Salerno [20] found no differences. Interestingly, Salerno reported that lower CASK scores among mothers, but not fathers, predicted poorer child outcomes at six months.

Taken together, previous studies support the CASK’s reliability and its value for assessing caregiving skills and evaluating interventions across different European cultures. Validations of the English, Spanish, and German versions confirmed the six-factor structure. However, despite the Dutch version being used in both research and clinical settings, there is as yet no validation study of the Dutch version of the CASK. Moreover, there is currently no cross-country research on care-givers skills in those caring for someone with an ED, yet this may be worthwhile to enhance sample sizes, allow for stronger and more reliable results, and examine cultural differences and similarities. This in turn may inform interventions for carers. Validating the Dutch version will allow for future cross-country research on care-givers skills. The present study aimed to validate the Dutch CASK in carers of adolescents with EDs, testing the six-factor model, examining internal consistency of the total scale and subscales, and exploring potential gender differences in perceived caregiving skills. Based on the original scale development [16] and existing Spanish and German validations [24, 27], we hypothesized that the Dutch version of the CASK would replicate the established six-factor structure. Furthermore, we expected the internal consistency of both the total score and the six subscales to be adequate. Finally, we hypothesize no differences on the CASK total and subscales for female versus male carers.

## Methods

### Participants and procedure

The study participants were carers of adolescents receiving services at Altrecht Eating Disorders Rintveld, a tertiary specialized treatment center in the Netherlands. Participants were recruited from two distinct clinical pathways: carers of those scheduled for an initial assessment (*n* = 209) and those already receiving treatment (*n* = 39). For the initial assessment group, completing the CASK was part of an online preparatory module designed to support the procedure. Data were collected between August 2020 and October 2022. Completion was a prerequisite for the initial assessment, unless undue burden or clinical urgency required exemption. Carers received an information letter and provided written informed consent for the use of their data for research. A brief, supplementary recruitment period in early 2022 involved therapists informing carers of adolescents already in treatment about the study. There were no specific criteria concerning the length of treatment. This supplementary effort yielded the additional 39 responses via a paper-based CASK. Exclusion criteria included insufficient Dutch proficiency and caregiving for an adolescent with suspected Avoidant/Restrictive Food Intake Disorder (ARFID), as this condition requires skills different from those assessed by the CASK. Given the typical patient population and the exclusion of ARFID, the majority of participants were most likely carers of an adolescent with AN. Since completion of the questionnaire was a prerequisite for admission to the initial assessment, the current sample is considered representative of the population of carers seeking help at our specialized treatment center. Notably, the entire recruitment period coincided with the COVID-19 pandemic. Consequently, it is probable that treatment capacity limitations led to extended waiting times for carers and adolescents awaiting initial assessment.

Of the 248 caregivers who completed the CASK, 139 were female and 109 were male. The mean age of the total carers sample was 48.3 years (SD = 4.7). Female carers had a mean age of 47.2 years (SD = 4.2), while male carers had a mean age of 49.7 years (SD = 4.8). In 85 cases (68.5%), both parents of the same child completed the questionnaire. In addition, 54 women and 24 men participated individually, without a corresponding questionnaire from the other carer. The children for whom the CASK was completed had a mean age of 14.8 years (SD = 1.5), with ages ranging from 10 to 18 years. The Medical Research Ethics Committee of the University Medical Center Utrecht reviewed the study protocol and concluded that it did not fall under the scope of the Medical Research Involving Human Subjects Act (WMO), and therefore no further ethical approval was required. The study was approved by the Institutional Review Board of Altrecht Mental Health Institute.

### Instrument

The original English version of the CASK [16] consists of 27 items describing caregiving situations related to EDs, rated on a visual analogue scale from 0 to 100. Respondents indicate their level of confidence in applying specific caregiving skills during the past week, with higher scores reflecting greater perceived competence. The questionnaire is structured around six distinct domains: Implementing bigger-picture thinking and not getting caught up in the details of the illness (Bigger Picture, 7 items), strategies to improve carers’ own mood and resilience (Self-Care, 4 items), not getting caught up in nagging and bickering about the illness (Biting-Your-Tongue, 3 items), the ability to recognize symptoms as part of the illness (Insight and Acceptance, 3 items), the ability to regulate emotional reactions, despite being provoked, the ability to discuss emotions and to have empathy for the other (Emotional Intelligence, 5 items), and the ability to withhold getting drawn into conflict about aspects of the illness (Frustration Tolerance, 5 items) .

The translation process from English to Dutch was carried out in multiple steps. First, the original questionnaire was translated into Dutch by a certified medical translation agency. Next, the Dutch version was back-translated into English by a bilingual psychologist with extensive expertise in the field of ED (last author, LS). Discrepancies between the original and the back-translated versions were carefully reviewed and resolved through discussion and consensus between the researcher and the back-translator. For a few selected items, we consulted an experienced local clinician to align the terminology with the specific treatment concepts used in Dutch clinical practice. For example, the final Dutch version used the term ‘agreed-upon goals’ for the original concept of ‘behaviour targets’ (Item 18), and used the phrase ‘giving compliments’ for the term ‘praise’ (Item 17). The Dutch version of the CASK is provided in the Supplementary Materials.

### Statistical analysis

A Confirmatory Factor Analysis (CFA) was conducted using the lavaan package in R to verify the original structure of the questionnaire. The analysis employed maximum likelihood estimation. As recommended by Hu and Bentler [28], model fit was assessed using root mean squared error of approximation (RMSEA), comparative fit index (CFI), Tucker-Lewis index (TLI), and standardized root mean squared residual (SRMR). Consistent with established guidelines, acceptable model fit was determined by the following criteria: CFI ≥ 0.90, TLI ≥ 0.90, RMSEA ≤ 0.08, and SRMR ≤ 0.08. In case the initial model did not show acceptable fit, model modifications were explored in an exploratory post hoc analysis.

In case model fit of the overall six-factor structure proved inadequate, additional exploratory CFAs were conducted at the subscale level. Each subscale was tested separately as an unidimensional model to assess whether the hypothesized item grouping showed acceptable fit when evaluated independently. Model fit was again assessed using CFI, TLI, RMSEA, and SRMR.

Internal consistency of the subscales and the total scale was assessed using McDonald’s omega (ω), which provides a model-based estimate of reliability based on the factor loadings [29]. A threshold of ω ≥ 0.70 was considered indicative of acceptable reliability.

To examine differences in CASK total scores between male and female carers, a linear mixed-effects model (LMM) was performed using the lmerTest package in R. Given that 68% of the sample consisted of dyads (partners within the same family), a standard t-test would violate the assumption of independent observations. To account for this non-independence and the hierarchical structure of the data, a random intercept for dyad membership was included in the model. In this model, caregiver gender was entered as a fixed effect, while the dyadic grouping was treated as a random effect. This approach allows for the estimation of the effect of caregiver gender while controlling for the shared variance within families. Degrees of freedom and p-values were estimated using Satterthwaite’s approximation.

## Results

### Descriptive statistics of the CASK scale

The descriptive statistics for the CASK total score and its subscales across the total sample (*N* = 248) are presented in Table 1. This table summarizes the mean scores, standard deviations (SD), and the distribution of scores including the median and quartiles (Q1 and Q3).

**Table 1:** Descriptive statistics of CASK total and subscale scores

	Mean	SD	Median	Q1	Q3	Min.	Max.
CASK total score	68.11	11.81	67.41	60.37	75.83	39.26	100
Bigger Picture	75.13	13.00	75.71	65.71	84.29	41.43	100
Self-Care	58.39	18.74	60.00	42.50	72.50	7.50	100
Biting-Your-Tongue	58.05	17.75	56.67	46.67	70.00	10.00	100
Insight and Acceptance	69.14	16.25	70.00	57.50	80.00	20.00	100
Emotional Intelligence	70.49	16.34	72.00	60.00	82.00	16.00	100
Frustration Tolerance	69.13	14.04	72.00	60.00	80.00	30.00	100

Values are presented as mean and standard deviation (SD). Median scores and the first (Q1) and third (Q3) quartiles are provided to show the distribution

### Factorial validity and reliability of the CASK and the subscales

A confirmatory factor analysis (CFA) was conducted to test the originally proposed six-factor structure of the questionnaire. Model fit indices indicated insufficient fit to the data: CFI = 0.774, TLI = 0.744, RMSEA = 0.090, and SRMR = 0.090. Based on these results, further post hoc model adjustments were explored to improve fit. In a first exploratory step, residual correlations were added, allowing for covariance among their unique variances. This modified model showed a slight improvement in fit: CFI = 0.792, TLI = 0.746, RMSEA = 0.090, and SRMR = 0.080. In a second post hoc analysis, modification indices were used to identify additional model misfit. Several residual covariances between item error terms were added. This final, post hoc modified model demonstrated improved fit: CFI = 0.846, TLI = 0.819, RMSEA = 0.076, and SRMR = 0.074. On absolute fit indices (RMSEA and SRMR), the model demonstrated acceptable fit, but still suboptimal fit according to incremental fit indices (CFI and TLI). While these results suggest an improved model compared to the initial specification, the overall fit remains below conventional standards for a well-fitting model.

Given the suboptimal fit of the overall six-factor model, follow-up confirmatory factor analyses were conducted on each of the six subscales separately to examine whether the individual constructs demonstrated acceptable unidimensional structure. Most subscales showed excellent incremental fit, with CFI and TLI values exceeding the commonly accepted threshold of 0.95. However, absolute fit as indicated by RMSEA was mixed. This discrepancy was particularly evident in the shorter subscales (Self-Care, Biting-Your-Tongue, Insight and Acceptance), which contained three to four items and exhibited inflated RMSEA values (≥ 0.085), a known limitation of this index in models with low degrees of freedom [30]. SRMR values were within acceptable limits for all subscales, suggesting no major localized misfit. The internal consistency of the multi-item scales was generally good, with five of the six subscales demonstrating strong reliability coefficients (ranging from ω = 0.78 to ω = 0.85). One subscale, Insight and acceptance, reported a coefficient (ω = 0.68) that falls just below the conventional 0.70 threshold. While this value is deemed marginally acceptable for group-level research, it signals a lower measurement precision for this specific construct. Fit indices and McDonald’s omega (ω) values are presented in Table 2. The standardized factor loadings of the original six factor model and the subscale models are presented in Table S1a and S1b of the Supplementary Materials. To further visualize the relationships between the items and their respective latent factors, graphical model plot have also been included in figure S1 and S2 of the Supplementary Materials.

**Table 2:** Reliability and model fit indices of the subscales of the CASK

Subscale	Number of items	CFI	TLI	RMSEA	SRMR	ω
Bigger Picture	7	0.992	0.988	0.082	0.051	0.85
Self-Care	4	0.994	0.982	0.138	0.044	0.80
Biting-Your-Tongue	3	0.997	0.996	0.085	0.032	0.82
Insight and Acceptance	3	1.000	1.011	0.000	0.011	0.68
Emotional Intelligence	5	0.964	0.929	0.222	0.085	0.83
Frustration Tolerance	5	0.998	0.995	0.054	0.054	0.78

*CFI* Comparative Fit Index, *TLI* Tucker–Lewis Index, *RMSEA* Root Mean Square Error of Approximation, *SRMR* Standardized Root Mean Square Residual. Conventional cutoffs: CFI/TLI > 0.90 = acceptable, > 0.95 = good; RMSEA < 0.08 = acceptable, < 0.06 = good; SRMR < 0.08 = acceptable. Fit interpretation is based on these guidelines. ω = McDonald’s omega. Conventional cutoffs: ω > 0.70 = acceptable, > 0.80 = good

### Group differences between female and male carers

There were no significant differences between female and male carers on the total CASK score, indicating comparable self-reported caregiving skills across genders. However, several subscale differences emerged. Male carers reported significantly higher skills on the Self-Care subscale and the Insight and Acceptance subscale compared to female carers. Conversely, female carers reported significantly higher skills on the Frustration Tolerance subscale and the Emotional Intelligence subscale. No significant differences were observed for the Bigger Picture and Biting-Your-Tongue subscales. The intraclass correlation coefficients (ICC) ranged from 0.23 to 0.46 across the models, indicating that a substantial proportion of the variance (23% to 46%) was accounted for by dyad membership. See Table 3.

**Table 3:** Comparison of cask scores between female and male carers

	Female carers (*n* = 139)	Male carers (*n* = 109)	B(Estimate)	SE	df	t	*p*-value	ICC	Cohen’s d
CASK total score	68.22 (12.07)	68.04 (11.53)	0.36	1.28	127.29	0.28	0.780	0.36	0.03
Bigger Picture	76.11 (13.28)	73.87 (12.57)	2.14	1.51	135.56	1.42	0.158	0.24	0.16
Self-Care	54.78 (19.33)	62.98 (16.96)	-7.89	1.85	124.10	-4.26	< 0.001	0.46	-0.43
Biting-Your-Tongue	58.23 (18.36)	57.83 (17.03)	0.62	2.03	136.65	0.30	0.762	0.28	0.03
Insight and Acceptance	66.31 (16.85)	72.76 (14.75)	-6.55	1.87	130.13	-3.51	0.001	0.23	-0.41
Emotional Intelligence	72.07 (15.25)	68.48 (17.51)	3.92	1.88	125.58	2.08	0.039	0.25	0.24
Frustration Tolerance	71.21 (12.96)	66.48 (14.96)	4.89	1.56	134.55	3.13	0.002	0.29	0.35

Values are presented as Mean and Standard Deviation (SD). Observations nested within 163 dyads. Model fit using Restricted Maximum Likelihood (REML) with Satterthwaite’s approximation for degrees of freedom (df). B represents the fixed effect estimate for sex; a negative value indicates higher scores for male carers, while a positive value indicates higher scores for female carers. ICC = Intraclass Correlation Coefficient, representing the proportion of variance at the dyad level. Effect sizes are reported using Cohen’s d

## Discussion

This study aimed to validate the Dutch translation of the Caregiver Skills scale (CASK) in a sample of carers who had presented to a tertiary specialized eating disorder treatment center seeking care for an individual with an ED. Furthermore, this study aimed to examine gender differences in self-reported caregiving skills.

The original six-factor structure did not show acceptable global fit in the confirmatory factor analysis (CFA). Although post-hoc modifications—such as freeing error covariances—resulted in improved fit indices, the overall model remained below conventional thresholds for a well-fitting structure. In contrast, analyses at the subscale level demonstrated excellent incremental fit and acceptable absolute fit (SRMR within limits), supporting the independent use of these subscales. The internal consistency of the subscales was generally high, supporting the reliability of the instrument in this population. However, the Insight and Acceptance subscale fell just short of the 0.70 threshold. Overall, the findings suggest that the CASK may be informative at a group level, namely for evaluating caregiver interventions and other research purposes. Secondly, our data suggest that the CASK may be relevant for informing policies concerning support strategies and required resources for those caring for someone with an ED. Our data does not allow drawing conclusions on the usefulness of the CASK for individual clinical decision-making, e.g. for informing individualized treatment content [31].

The findings regarding the six-factor structure contrast with previous validation studies of the CASK conducted in Spanish and German translations. Vintro-Alcaraz et al. (2018) validated the CASK in a sample of 265 caregivers (63% females) of individuals with ED (mean age 19.1 years), with about half meeting criteria for AN and the other half being diagnosed with bulimia nervosa (BN), binge eating disorder (BED), or Other Specified Feeding or Eating Disorder (OSFED). They found a clear replication of the original six-factor structure using confirmatory factor analysis (CFA), supporting the questionnaire’s theoretical model without substantial modifications. Internal consistency was moderate across subscales [27]. In contrast, the German validation by Zeiler et al. [24], performed in a sample of 233 caregivers (76% female) caring for adolescents with AN (mean age = 15.1 years) initially failed to confirm the original factor structure and required several statistical adjustments—such as freeing error covariances based on modification indices—to achieve acceptable model fit. Internal consistency was excellent for the total scale and ranged from acceptable to good across subscales [24]. A very recently published paper reports a poor fit for the Italian version of the CASK one-factor solution, highlighting the multidimensionality of the construct [32].

The reasons for the discrepancies between our findings and those of previous validation studies are not immediately clear and may relate to a combination of linguistic and contextual factors. Compared to the Spanish [27] and German [24] studies, our study shares several key similarities. Sample sizes were broadly comparable, and the mean age of carers in our study fell within the same range as those reported in the earlier validations. Although we did not collect information on carers’ education level, there is no indication that the educational background of our sample differed substantially from that of participants in the Spanish or German studies (which were typically highly educated). At the same time, there were several notable differences that may have influenced our findings. First, our sample had a more balanced gender distribution among carers (56% female), whereas previous studies included relatively more female participants (64–76%). Second, The mean age of the adolescents cared for in our study (14.8 years of age) was younger than that in the Spanish study (19.1 years of age [27]), although it did not substantially differ from the German study (15.1 years of age [24]). Third, the timing of questionnaire administration differed: in our study, most caregivers completed the CASK prior to assessment and the start of treatment, whereas in the Spanish study [27] participants were already engaged in ED support groups, and in the German study [24], they had either taken part in or expressed willingness to attend caregiver interventions. These carers were therefore likely more exposed to therapeutic language and concepts, had more insight into their caregiving roles, and/or felt more confident in their skills. Indeed, the Spanish study reported higher average levels of self-reported skills (female carers: 73.1; male carers 75.5) compared to our study (male and female caregivers combined 68.2). However, the skills reported in the German study were largely similar to those found in our sample (male and female caregivers combined 68.5).

Although it is unclear whether, and to what extent, these differences between studies can explain the disparate outcomes, it is conceivable that these variations may have influenced how carers interpreted and responded to the questionnaire items. Consequently, this had potentially influenced relationships between items, contributing to the different factor structures observed across the studies. A certain familiarity with ED terminology and treatment processes may be important for an accurate understanding of the questionnaire items, and may also enable carers to provide a more valid self-assessment of their skills. In fact, one of the main questions following this and other CASK studies is at what stage during the illness would the CASK be most appropriate and valid to use? On the one hand it may be in a later stage, when carers have been familiarized with ED language and their own behaviours and are thus more able to recognize their own (lack of) skills and the need to address these. At the other hand one could argue that the earlier in the illness we make parents aware of their skills and improve these, the better the long term illness prognoses could be. Hopefully future research into carers skills aims to address this question.

In our study, male and female carers did not differ in their overall CASK total scores, suggesting comparable self-perceived overall caregiving competence across genders. However, differences emerged at the subscale level. Male carers reported significantly higher scores on Self-Care and Insight and Acceptance. This indicates that male carers may perceive themselves as more able to balance caregiving demands with personal and family needs and to recognize symptoms as part of the illness. It is important to note that the reliability for the Insight and Acceptance subscale was slightly below the optimal threshold (ω = 0.68). Consequently, the findings pertaining to this specific subscale should be interpreted with an appropriate degree of caution. In contrast, female carers scored significantly higher on Frustration Tolerance and Emotional Intelligence, indicating a stronger self-perceived capacity to remain calm and empathetic in emotionally charged situations. Specifically, these findings suggest that female carers may perceive themselves as more adept at regulating emotional reactions despite being provoked and maintaining the ability to discuss emotions and empathize with the adolescent with an ED. This pattern of results partially aligns with earlier studies. With respect to the total score, the Spanish study [27] also reported no significant gender differences, contrasting with the German study [24], which found that men reported stronger overall caregiving skills than women.

Regarding the subscales, the Spanish validation study [27] found no significant gender differences in the entire sample, suggesting broadly comparable self-perceptions of caregiving skills across male and female carers in that sample. However, looking at a subsample that included only carers whose partner had also provided data (for the same child), a slightly different picture emerged, with fathers scoring higher on The Bigger Picture and Biting-Your-Tongue subscales compared to the mothers [27]. Indeed, the German study [24] also reported that male carers scored higher on Biting-Your-Tongue. This study furthermore reported that, in line with our study, male carers scored higher on Self-Care and Insight and Acceptance. These differences between female and male carers may reflect a greater tendency among male carers to perceive themselves as better able to regulate their emotions and maintain boundaries and a greater capacity to avoid unproductive conflict in caregiving situations. As also suggested by Vintró-Alcaraz et al. [27], female carers may be more emotionally involved in the illness and therapy process and be less able to keep a life span perspective rather than a focus on detail, which in turn may reduce their ability to remain emotionally more distant. As highlighted by both Vintró-Alcaraz et al. [27] and Pehlivan et al. [4], it is also possible that differing personality traits between female and male carers play a role. For example, higher levels of anxiety, depression, distress an burden of caring have been found in female carers compared to male carers [26, 33].

Salerno et al. [20] found no difference on CASK scores between female and male caregivers. However, lower CASK scores for female caregivers was related to poorer ED outcomes of their child at six months, whilst male caregivers’ CASK scores were not related to outcomes. Taking together our study and the earlier studies, there is currently insufficient evidence to draw firm conclusions about systematic gender differences in CASK scores. Nonetheless, the subscale differences observed may offer preliminary insights for clinical practice. These results tentatively suggest that female carers might benefit from an increased focus on self-care and personal boundaries, whereas male carers could potentially gain from support aimed at strengthening emotional attunement and navigating interpersonal tension. Given the inconsistencies in the literature, these gender-specific patterns should be considered explorative rather than definitive.

Several limitations of this study should be acknowledged. First, although the sample size is comparable to those in previous validation studies of the CASK, it may be underpowered for robustly testing the factor structure of a 27-item instrument with six subscales. Second, the current study focused primarily on structural validity and internal consistency. Other forms of validation—such as convergent and discriminant validity, test–retest reliability and sensitivity for change—were not assessed. Third, the majority of the caregivers (*N* = 209) completed the questionnaire online. While in earlier studies the administration methods were not explicitly detailed, the transition to digital platforms warrants consideration. However, as comparative psychometric research generally indicates that the measurement properties of self-report scales remain stable and comparable across online and paper-based modes [34], any potential influence of the online format on the current results is expected to be minimal. Fourth, ED diagnoses were not systematically collected (except for excluding ARFID diagnoses). As most adolescents were likely diagnosed with AN, it remains unclear whether findings generalize to other ED diagnoses. Future research in more diagnostically diverse samples is needed. For example, all the (published) CASK studies include carers from Western-European countries, and future research should focus on including non Western-European countries, where the norms and values of care-taking, but also the degree of acceptability that one’s own behaviours can be more or less helpful, may be very different from Western European norms and values.

## Conclusions

The Dutch version of the CASK demonstrates sufficient psychometric quality to support its use in research contexts, including group-level comparisons and the evaluation of interventions for carers. Additionally, it may inform policies aimed at optimizing support strategies and resources for carers. Both the total score and the individual subscales can be used to capture meaningful differences and changes in reported caregiver skills at the group level. In other words, the CASK can be a valuable tool to assess and improve caregiving interventions. These are much needed seeing that carers for someone with an ED report high levels of psychological distress and carers burden, alongside feelings of inadequacy to perform the often complex caring responsibilities. They require more information about EDs and treatments, and more guidance in their caregiving role [4–6]. Importantly, caregiving skills are associated to treatment outcomes in that the adolescents with AN whose carers had taken part in a carers intervention had higher rates of recovery compared to those whose carers did not take part in the intervention [7]. Given the limited fit of the six-factor model, the instrument is, at this stage, not recommended for use in individual clinical decision-making. Our findings also suggest potential gender differences in self-perceived caregiving skills, particularly in domains such as Self-Care and Frustration Tolerance. While these trends are consistent with observations in the German validation study, the current evidence base remains limited. Further research is therefore needed to investigate gender-related response patterns. Other important and interesting avenues for future research include examining the utility of the CASK for specific caregiving subgroups, such as parents of young children and carers of children with co-occurring conditions, including autism spectrum disorder. Such a nuanced understanding of diverse caregiver populations is crucial for developing and implementing tailored, needs-based interventions.

## Supplementary Information

Below is the link to the electronic supplementary material.

Supplementary Material 1.

Supplementary Material 2.

Supplementary Material 3.

## Data Availability

Data supporting the findings of this study are available from the corresponding author upon reasonable request.

## Abbreviations

AN: Anorexia nervosa
ARFID: Avoidant/restrictive food intake disorder
BED: Binge eating disorder
BN: Bulimia nervosa
CASK: Caregiver skills scale
CFA: Confirmatory factor analysis
CFI: Comparative fit index
ED(s): Eating disorder(s)
FBT: Family-based treatment
MFT: Multi-family therapy
OSFED: Other specified feeding or eating disorder
RMSEA: Root mean square error of approximation
SD: Standard deviation
SRMR: Standardized root mean square residual
TLI: Tucker-lewis index
WMO: Wet Medisch-Wetenschappelijk Onderzoek (Medical Research Involving Human Subjects Act)
ω: McDonald’s Omega (Internal Consistency Index)

## Declarations

All participating carers received an information letter detailing the study’s purpose and procedures. Written informed consent was obtained from all carers who agreed to the use of their completed questionnaires for the study.
