# Supplementary material for: Validation of the caregiver skills (CASK) scale in a Dutch sample of carers for adolescents with eating disorders
Source: J Eat Disord. 2026 Mar 2;14:77. doi: 10.1186/s40337-026-01561-6 (PMC13059607; doi:10.1186/s40337-026-01561-6)
Supplement: Supplementary file 1 — Supplementary Material 1. [file 40337_2026_1561_MOESM1_ESM.pdf]

Caregiver Skills (CASK, vaardigheden van zorgverleners)

We zijn benieuwd naar uw mening over bepaalde onderwerpen met betrekking tot de zorg voor uw kind. We willen u vragen om zo eerlijk en openhartig mogelijk te zijn.

Hieronder worden situaties beschreven die doorgaans worden geassocieerd met eetstoornissen. Geef voor elke situatie aan hoe zeker u weet dat u in staat zou zijn om te reageren zoals omschreven.

Gebruik onderstaande beoordelingsschaal om die zekerheid te waarderen met een score tussen 0 en 100.

0 10 20 30 40 50 60 70 80 90 100
Vrijwel nooit Soms Vaak Vrijwel altijd

Een score van 100 betekent bijvoorbeeld dat u 100% zeker weet dat u de activiteit op elk gewenst moment kunt uitvoeren. Omcirkel bij elk scenario het cijfer dat uw gevoel van zekerheid het beste weergeeft. Maak een keuze uit een score tussen 0 en 100 (10, 20, 30 enz.).

Baseer uw scores op wat u DEZE WEEK kunt betekenen als de persoon die u NU bent en niet als degene die u was of zou willen zijn. Dit is zeer belangrijk.

Hebt u het gevoel dat sommige vragen niet op u van toepassing zijn? Probeer te beoordelen hoe zeker u zich zou voelen als een bepaalde situatie zich zou voordoen.

De witruimte verwijst naar uw dierbare met een eetstoornis. Deze ruimte hoeft niet te worden ingevuld.

Hartelijk dank voor het invullen van deze enquête.

Hoe zeker weet u dat u in staat zou zijn...

1. … De dingen te blijven doen die u leuk vindt terwijl u zorgt voor ________?

   0 10 20 30 40 50 60 70 80 90 100
   Vrijwel nooit Soms Vaak Vrijwel altijd

2. … Uw eigen gevoelens over de eetstoornis openhartig toe te lichten en te bespreken met _________?

   0 10 20 30 40 50 60 70 80 90 100
   Vrijwel nooit Soms Vaak Vrijwel altijd

3. ….. De eetstoornis openhartig te bespreken met *alle* andere betrokken gezinsleden?

   0 10 20 30 40 50 60 70 80 90 100
   Vrijwel nooit Soms Vaak Vrijwel altijd

4. ….. Begrip te kunnen opbrengen voor _______, ook als u boos op, of teleurgesteld in_______ bent?

0 10 20 30 40 50 60 70 80 90 100
Vrijwel nooit Soms Vaak Vrijwel altijd

5. ….. Te kunnen voorkomen in een ruzie te verzeilen met _________ over de eetstoornis?

0 10 20 30 40 50 60 70 80 90 100
Vrijwel nooit Soms Vaak Vrijwel altijd

6. ….. Kalm te blijven als u wordt geconfronteerd met lastig gedrag dat wordt veroorzaakt door de eetstoornis?

0 10 20 30 40 50 60 70 80 90 100
Vrijwel nooit Soms Vaak Vrijwel altijd

7. ….. Een moment voor uzelf te nemen als u daar behoefte aan hebt?

0 10 20 30 40 50 60 70 80 90 100
Vrijwel nooit Soms Vaak Vrijwel altijd

8. ….. Met _________ te praten en te luisteren naar de moeilijke en gecompliceerde gevoelens die hij/zij ervaart?

0 10 20 30 40 50 60 70 80 90 100
Vrijwel nooit Soms Vaak Vrijwel altijd

9. …. Zich gerust te laten stellen door zelfs de kleinste tekenen van verbetering?

0 10 20 30 40 50 60 70 80 90 100
Vrijwel nooit Soms Vaak Vrijwel altijd

10. ….. Hoop te blijven houden dat _______ zal herstellen?

0 10 20 30 40 50 60 70 80 90 100
Vrijwel nooit Soms Vaak Vrijwel altijd

11. ….. Een stap terug te doen en erop te vertrouwen dat ____________ zelf kan omgaan met dagelijkse uitdagingen?

0 10 20 30 40 50 60 70 80 90 100
Vrijwel nooit Soms Vaak Vrijwel altijd

12. … Samen met _________ grenzen te stellen, plannen te maken en huisregels op te stellen?

0 10 20 30 40 50 60 70 80 90 100
Vrijwel nooit Soms Vaak Vrijwel altijd

13. … Consistent en op meelevende wijze vast te houden aan grenzen/regels, ook als ________ het daar niet mee eens is?

0 10 20 30 40 50 60 70 80 90 100
Vrijwel nooit Soms Vaak Vrijwel altijd

14. … De behoefte te onderdrukken om in discussie te gaan over het gedrag dat bij de eetstoornis hoort, ook als u denkt dat u gelijk hebt?

0 10 20 30 40 50 60 70 80 90 100
Vrijwel nooit Soms Vaak Vrijwel altijd

15. ….. Goede gesprekken te kunnen voeren met ___________ die geen betrekking hebben op de eetstoornis?

0 10 20 30 40 50 60 70 80 90 100
Vrijwel nooit Soms Vaak Vrijwel altijd

16. ….. De behoefte te onderdrukken om te vragen of te informeren naar het gedrag van _______, ook als u zich zorgen maakt?

0 10 20 30 40 50 60 70 80 90 100
Vrijwel nooit Soms Vaak Vrijwel altijd

17. ….. Complimenten geven voor verandering of pogingen tot verandering door _______, ook als het effect/resultaat minder groot is dan gehoopt?

0 10 20 30 40 50 60 70 80 90 100
Vrijwel nooit Soms Vaak Vrijwel altijd

18. ….. De behoefte te onderdrukken om voortdurend terug te komen op afgesproken doelen?

0 10 20 30 40 50 60 70 80 90 100
Vrijwel nooit Soms Vaak Vrijwel altijd

19. ….. Te voorkomen dat u dezelfde gesprekken over voedsel en eten blijft voeren met _________?

0 10 20 30 40 50 60 70 80 90 100
Vrijwel nooit Soms Vaak Vrijwel altijd

20. ….. Het grotere geheel/de algemene vooruitgang van ________ voor ogen te houden?

0 10 20 30 40 50 60 70 80 90 100
Vrijwel nooit Soms Vaak Vrijwel altijd

21. ….. De behoefte te onderdrukken om uitsluitend op basis van het gewicht te bepalen hoe hij/zij het doet?

0 10 20 30 40 50 60 70 80 90 100
Vrijwel nooit Soms Vaak Vrijwel altijd

22. ….. ___________ als persoon los te koppelen van de ziekte?

0 10 20 30 40 50 60 70 80 90 100
Vrijwel nooit Soms Vaak Vrijwel altijd

23. …. Na te denken over uw gedrag en te begrijpen wat het effect daarvan is op __________?

0 10 20 30 40 50 60 70 80 90 100
Vrijwel nooit Soms Vaak Vrijwel altijd

24. ….. Te accepteren dat de eetstoornis niet uw schuld is?

0 10 20 30 40 50 60 70 80 90 100
Vrijwel nooit Soms Vaak Vrijwel altijd

25. ….. Te accepteren dat de oorzaak of aanleiding voor de eetstoornis mogelijk niet de oplossing voor herstel is?

0 10 20 30 40 50 60 70 80 90 100
Vrijwel nooit Soms Vaak Vrijwel altijd

26. … Ruimte te maken om tijd door te brengen met andere gezinsleden?

0 10 20 30 40 50 60 70 80 90 100
Vrijwel nooit Soms Vaak Vrijwel altijd

27. … Uw angst onder controle te hebben, zodat u zich niet overweldigd voelt?

0 10 20 30 40 50 60 70 80 90 100
Vrijwel nooit Soms Vaak Vrijwel altijd

Hartelijk dank voor het voltooien van deze enquête.
