# Supplementary material for: Validation of the caregiver skills (CASK) scale in a Dutch sample of carers for adolescents with eating disorders
Source: J Eat Disord. 2026 Mar 2;14:77. doi: 10.1186/s40337-026-01561-6 (PMC13059607; doi:10.1186/s40337-026-01561-6)
Supplement: Supplementary file 2 — Supplementary Material 2. [file 40337_2026_1561_MOESM2_ESM.docx]

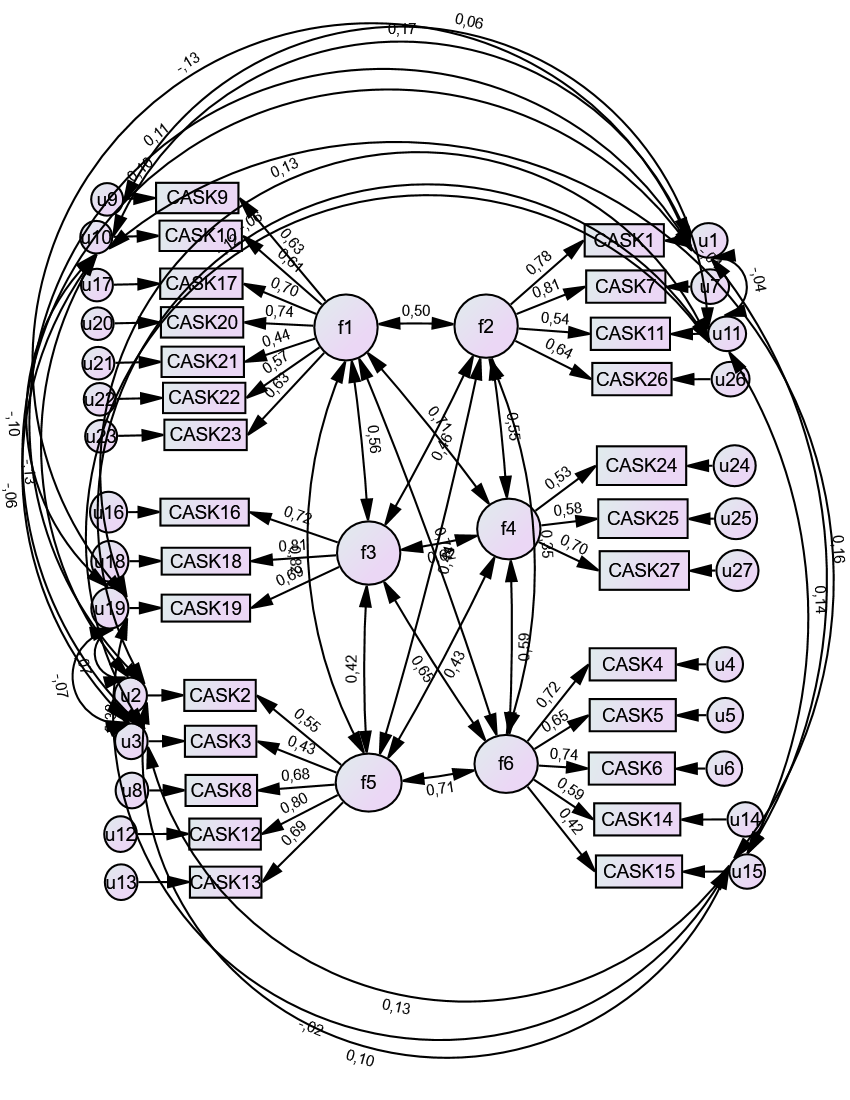


**Figure S1.** Path diagram of the modified 6-factor model of the CASK including factor loadings and correlations between latent factors. f1 Bigger Picture, f2 Self-Care, f3 Biting your Tongue, f4 Insight & Acceptance, f5 Emotional Intelligence, f6 Frustration Tolerance


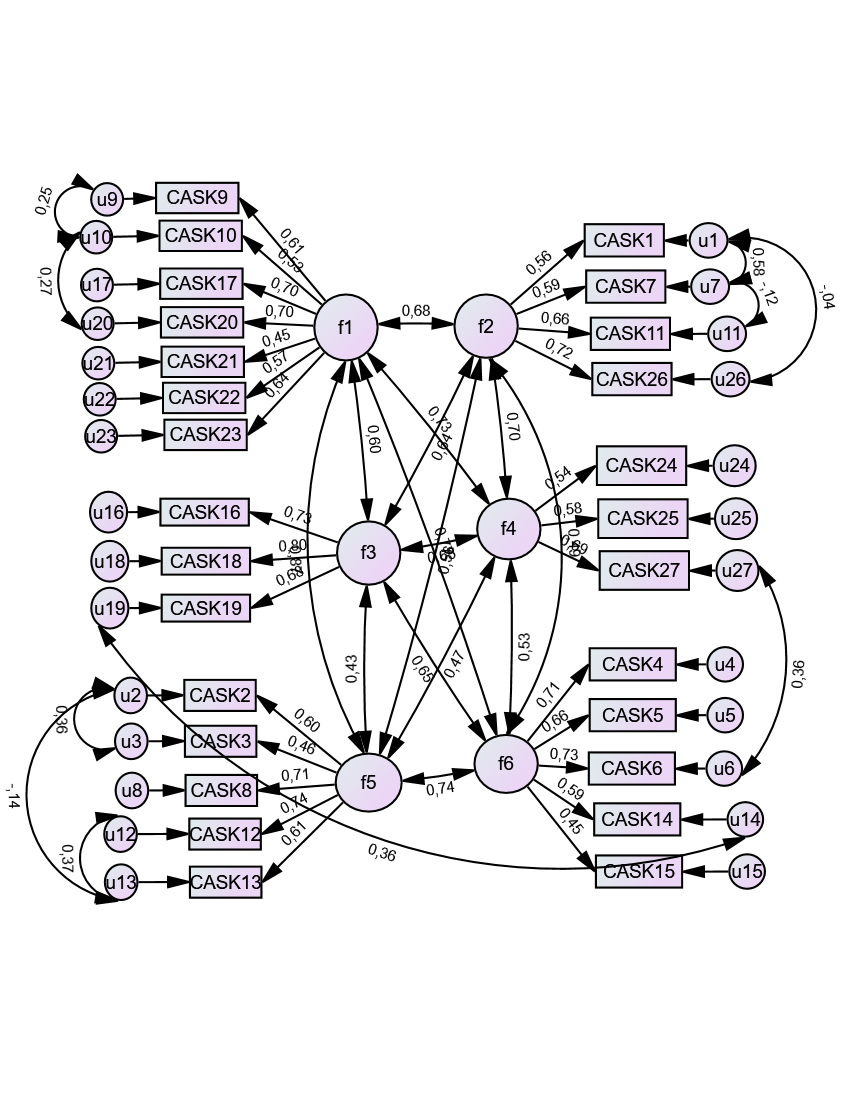
**Figure S2.** Path diagram of the 6-factor model of the CASK including factor loadings and correlations between latent factors. f1 Bigger Picture, f2 Self-Care, f3 Biting your Tongue, f4 Insight & Acceptance, f5 Emotional Intelligence, f6 Frustration Tolerance
