# Supplementary material for: Validation of the caregiver skills (CASK) scale in a Dutch sample of carers for adolescents with eating disorders
Source: J Eat Disord. 2026 Mar 2;14:77. doi: 10.1186/s40337-026-01561-6 (PMC13059607; doi:10.1186/s40337-026-01561-6)
Supplement: Supplementary file 3 — Supplementary Material 3. [file 40337_2026_1561_MOESM3_ESM.docx]

**Table S1a Factor loadings for the Confirmatory Factor Analysis (CFA) of the original six-factor model**

| Factors and Items | CFA factor loadings | | | |
| --- | --- | --- | --- | --- |
|  | Estimate | SE | *Z-value* | P-value |
| *F1. Bigger Picture* |  |  |  |  |
| 09. Reassured by even small improv. | 0.637 | 0.061 | 10.459 | <.001 |
| 10. Keep hope that X will recover | 0.586 | 0.062 | 9.426 | <.001 |
| 17. Praise change or attempts | 0.701 | 0.059 | 11.860 | <.001 |
| 20. Keep your eye on X’s progress | 0.734 | 0.058 | 12.626 | <.001 |
| 21. Resist in relying solely on weight | 0.432 | 0.065 | 6.634 | <.001 |
| 22. Separate X as a person | 0.563 | 0.063 | 8.991 | <.001 |
| 23. Reflect and understand | 0.632 | 0.061 | 10.352 | <.001 |
| *F2. Self-Care* |  |  |  |  |
| 01. Keep doing things that you enjoy | 0.794 | 0.059 | 13.471 | <.001 |
| 07. Take some time for yourself | 0.811 | 0.059 | 13.843 | <.001 |
| 11. Step back and trust | 0.529 | 0.065 | 8.141 | <.001 |
| 26. Find time to spend with family | 0.634 | 0.063 | 10.118 | <.001 |
| *F3. Biting-Your-Tongue* |  |  |  |  |
| 16. Control urge enquiring checking | 0.720 | 0.061 | 11.785 | <.001 |
| 18. Resist constantly remind and ask | 0.807 | 0.059 | 13.623 | <.001 |
| 19. Avoid getting in conversations | 0.722 | 0.061 | 11.832 | <.001 |
| *F4. Insight and Acceptance* |  |  |  |  |
| 24. Accept that ED is not your fault | 0.532 | 0.069 | 7.677 | <.001 |
| 25. Insight there is no one cause | 0.579 | 0.069 | 8.449 | <.001 |
| 27. Manage your anxiety levels | 0.697 | 0.068 | 10.300 | <.001 |
| *F5. Emotional Intelligence* |  |  |  |  |
| 02. Discuss and explain feelings | 0.600 | 0.063 | 9.571 | <.001 |
| 03. Discuss the ED openly with family | 0.490 | 0.065 | 7.530 | <.001 |
| 08. Talk and listen with X emotions | 0.676 | 0.061 | 11.129 | <.001 |
| 12. Agree boundaries-plans | 0.793 | 0.057 | 13.783 | <.001 |
| 13. Uphold boundaries / rules | 0.673 | 0.061 | 11.063 | <.001 |
| *F6. Frustration Tolerance* |  |  |  |  |
| 04. Be understanding towards X | 0.713 | 0.060 | 11.830 | <.001 |
| 05. Avoid drawn into arguments | 0.647 | 0.062 | 10.436 | <.001 |
| 06. Be calm with difficult ED behavior | 0.731 | 0.060 | 12.222 | <.001 |
| 14. Control the urge to argue | 0.585 | 0.064 | 9.208 | <.001 |
| 15. Pleasant verbal interactions | 0.453 | 0.066 | 6.847 | <.001 |

Note. CFA = Confirmatory Factor Analysis. N=248. Standardized factor loadings (Estimate) and their corresponding Standard Errors (SE), Z-values, and p-values are presented for the final six-factor model. All estimates were obtained under full standardization (std.all). The model fit indices were: CFI = 0.774, TLI = 0.744, RMSEA = 0.090, and SRMR = 0.090.

**Table S1b Factor loadings for the Confirmatory Factor Analysis (CFA) of the subscales models**

| *F1. Bigger Picture* | CFA factor loadings | | | |
| --- | --- | --- | --- | --- |
|  | Estimate | SE | *Z-value* | P-value |
| 09. Reassured by even small improv. | 0.682 | 0.028 | 24.080 | <.001 |
| 10. Keep hope that X will recover | 0.671 | 0.035 | 19.347 | <.001 |
| 17. Praise change or attempts | 0.753 | 0.029 | 25.886 | <.001 |
| 20. Keep your eye on X’s progress | 0.839 | 0.022 | 37.292 | <.001 |
| 21. Resist in relying solely on weight | 0.484 | 0.040 | 11.961 | <.001 |
| 22. Separate X as a person | 0.590 | 0.039 | 15.069 | <.001 |
| 23. Reflect and understand | 0.625 | 0.039 | 16.075 | <.001 |

| *F2. Self-Care* | CFA factor loadings | | | |
| --- | --- | --- | --- | --- |
|  | Estimate | SE | *Z-value* | P-value |
| 01. Keep doing things that you enjoy | 0.822 | 0.031 | 26.506 | <.001 |
| 07. Take some time for yourself | 0.856 | 0.027 | 31.525 | <.001 |
| 11. Step back and trust | 0.530 | 0.041 | 12.786 | <.001 |
| 26. Find time to spend with family | 0.614 | 0.032 | 19.006 | <.001 |

| *F3. Biting-Your-Tongue* | CFA factor loadings | | | |
| --- | --- | --- | --- | --- |
|  | Estimate | SE | *Z-value* | P-value |
| 16. Control urge enquiring checking | 0.774 | 0.016 | 47.695 | <.001 |
| 18. Resist constantly remind and ask | 0.774 | 0.016 | 47.695 | <.001 |
| 19. Avoid getting in conversations | 0.774 | 0.016 | 47.695 | <.001 |

| *F4. Insight and Acceptance* | CFA factor loadings | | | |
| --- | --- | --- | --- | --- |
|  | Estimate | SE | *Z-value* | P-value |
| 24. Accept that ED is not your fault | 0.640 | 0.028 | 23.160 | <.001 |
| 25. Insight there is no one cause | 0.640 | 0.028 | 23.160 | <.001 |
| 27. Manage your anxiety levels | 0.640 | 0.028 | 23.160 | <.001 |

| *F5. Emotional Intelligence* | CFA factor loadings | | | |
| --- | --- | --- | --- | --- |
|  | Estimate | SE | *Z-value* | P-value |
| 02. Discuss and explain feelings | 0.707 | 0.032 | 21.978 | <.001 |
| 03. Discuss the ED openly with family | 0.604 | 0.038 | 15.815 | <.001 |
| 08. Talk and listen with X emotions | 0.700 | 0.030 | 22.969 | <.001 |
| 12. Agree boundaries-plans | 0.814 | 0.024 | 33.560 | <.001 |
| 13. Uphold boundaries / rules | 0.692 | 0.033 | 21.283 | <.001 |

| *F6. Frustration Tolerance* | CFA factor loadings | | | |
| --- | --- | --- | --- | --- |
|  | Estimate | SE | *Z-value* | P-value |
|  |  |  |  |  |
| 04. Be understanding towards X | 0.765 | 0.027 | 28.061 | <.001 |
| 05. Avoid drawn into arguments | 0.718 | 0.027 | 26.434 | <.001 |
| 06. Be calm with difficult ED behavior | 0.748 | 0.033 | 22.371 | <.001 |
| 14. Control the urge to argue | 0.563 | 0.031 | 18.079 | <.001 |
| 15. Pleasant verbal interactions | 0.406 | 0.046 | 8.766 | <.001 |

Note. CFA = Confirmatory Factor Analysis. N=248. Standardized factor loadings (Estimate) and their corresponding Standard Errors (SE), Z-values, and p-values are presented for the subscales
